# Investigating the neural architecture of handedness

**DOI:** 10.3389/fpsyg.2015.00148

**Published:** 2015-02-11

**Authors:** Sebastian Ocklenburg, Alexis Garland, Felix Ströckens, Anelisie Uber Reinert

**Affiliations:** Institute of Cognitive Neuroscience, Department of Biopsychology, Ruhr University BochumBochum, Germany

**Keywords:** handedness, genetics, cortex, ontogenesis, endophenotype, motor cortex, MRI imaging

When we look at our hands, outwardly they look remarkably similar to each other—and the bones, muscles and nerves within the two hands are equally symmetric. Way before the advent of modern neuroimaging, this fact led researchers to the conclusion that the striking preference most people show in using one hand over the other to conduct fine motor tasks must originate somewhere in the cerebral cortex and not in the peripheral nervous system (McManus, 1996). However, the question of where exactly has been proven surprisingly hard to answer, even with advanced neuroimaging methods. Functionally, the cortical correlates of handedness have been investigated by different authors using fMRI (functional magnetic resonance imaging), for example by measuring brain activity in motor areas of left- and right-handers during unilateral or bilateral finger or hand movements (e.g., Gut et al., 2007; Klöppel et al., 2007; Grabowska et al., 2012). In addition, several studies were aimed at identifying the macrostructural correlates of left-and right-handedness in the cerebral cortex (e.g., regarding the volume of different gray matter brain areas), with mixed results. While some authors found significant structural differences between left and right-handers, e.g., in the precentral gyrus and sulcus (Amunts et al., 1996; Foundas et al., 1998), the planum temporale (Hervé et al., 2006) and Broca's area (Powell et al., 2012a), others could not identify any macrostructural correlates of handedness (e.g., Good et al., 2001). These somewhat ambiguous results might in part be due to sample characteristics, since especially in studies with a small number of left-handers, individual anatomical properties in this group might have had a disproportionally large impact on the overall effect.

In the paper by Guadalupe et al. (2014), the authors avoided this issue by investigating an impressively large sample of 1960 right-handed and 106 left-handed participants, the largest cohort that has so far been investigated in relation to this question. In order to identify structural differences in the cortices of left- and right-handers, MRI-scans of the subjects were analyzed using an automated parcellation technique, which allowed for analysis of the cortical surface area of 74 different brain parcellations in each hemisphere. The authors then compared left- and right-handers regarding the cortical surface area of 10 different candidate regions related to language, motor control and visual processing which were obtained from previous studies investigating the structural correlates of handedness in the brain. While the authors found a nominally significant association between handedness and the surface area of the left precentral sulcus, not a single effect survived statistical correction for multiple testing. These striking results yield a very important insight into the structural correlates of handedness by showing that macrostructural properties of gray matter brain areas might not represent the major cortical correlate (or even “cause”) of handedness. Thus, the findings by Guadalupe et al. (2014) implicate that future research on structure-function relationship in regard to handedness should focus on other structural properties of the motor and related systems, e.g. the microstructure of gray matter areas (Chance, 2014) or micro- and macrostructural properties of white matter pathways (Powell et al., 2012b). Gaining a better understanding of the anatomical base of handedness is also of particular importance for several related areas within the broader scope of research, such as the ontogenesis and clinical significance of hemispheric asymmetries. For example, studies identifying structural markers for left- or right-handedness may provide endophenotypes that aid the ongoing quest to identify the genetic, epigenetic and environmental influences that determine handedness (e.g., Francks et al., 2007; Arning et al., 2013; Ocklenburg et al., 2013a). This in turn might benefit research investigating the ontogenesis of neuropsychiatric or developmental disorders related to a reduced frequency of right-handedness, such as schizophrenia (Hirnstein and Hugdahl, 2014), autism (Lindell and Hudry, 2013), and dyslexia (Brandler and Paracchini, 2014).

Aside from meta-analyses, single large-scale studies are one of the most important tools neuroscientists have for a comprehensive examination of structure-function relationships in the human brain (Hirnstein et al., 2014). Importantly, they avoid one of the biggest issues of meta-analyses: Due to the so-called file-drawer problem, e.g. the bias to only submit significant results to scientific journals, meta-analyses are strongly affected by publication biases (Rosenberg, 2005). By both avoiding this issue and the statistical power problems often associated with smaller studies, studies such as the work by Guadalupe et al. (2014) allow for comprehensive answers with regard to the association of structural properties of different brain regions and behavior. Hence, this type of study is exactly what is needed in order to advance our understanding of which properties of the brain are related to handedness and which are not. In this sense, a non-significant result in a large-scale study such as the one by Guadalupe et al. (2014) can be more meaningful than several contradicting significant findings in smaller, possibly underpowered, studies. In our opinion, this principle is not limited to handedness research but applies to any field of neuroscience and clinical research that heavily relies on groups comparisons between two or more groups showing high intra-group heterogeneity. For example, the field of cognitive sex differences (Hirnstein et al., 2013) or any type of patient-control comparison (Peterburs et al., 2012) e.g., between schizophrenic (Ocklenburg et al., 2013b) or autistic patients (Chmielewski and Beste, 2015) comes to mind.

We hope that the work by Guadalupe et al. (2014) encourages other researchers in cognitive neuroscience not only to conduct more studies with larger samples, but also to publish their findings even if they are non-significant.

## Conflict of interest statement

The authors declare that the research was conducted in the absence of any commercial or financial relationships that could be construed as a potential conflict of interest.
